# Shared practices among primary health care workers: A time-motion study

**DOI:** 10.1186/s12913-025-12439-9

**Published:** 2025-02-26

**Authors:** Talita Rewa, Geisa Colebrusco de Souza Gonçalves, Andrea Liliana Vesga Varela, Valéria Marli Leonello, Marina Peduzzi, Leticia Yamawaka de Almeida, Lorrayne Belotti, Debora Bernardo da Silva, Daiana Bonfim

**Affiliations:** 1 Hospital Israelita Albert Einstein, São Paulo, Brazil; 2 School of Nursing at, Sao Paulo University, USP, São Paulo, Brazil; 3 Universidade Federal de São Paulo, UNIFESP, São Paulo, Brazil

**Keywords:** Primary Health Care, Health Workforce, Interprofessional Relations, National Health Strategies, Unified Health System

## Abstract

**Background:**

Shared activities by health workers in meeting health needs are crucial to improve the health population, patient experience, quality of life of health teams members and the effectiveness of care.

**Objective:**

To identify the activities shared by Primary Health Care (PHC) team workers, whether multiprofessional or uniprofessional.

**Method:**

A descriptive study was, thus, carried out, based on the time-motion technique, in São Paulo-Brazil. Data collection was carried out from 2021 to 2022, through direct observations of workers, family health teams (FHT), oral health teams (OHT) and multiprofessional teams (MultiT). The frequency rates of both activities and time of shared practices and the distribution of workers involved in information exchanges were considered.

**Results:**

A total of 93 workers were observed, recording 21,936 activities (157,653 minutes). Of these, 72.90% were individual activities and 27.10%, shared (36.1% uni-professional and 63.9% multiprofessional). Shared activities represented 26.26% of the time. Dentists presented 60.8% of their activities in the shared modality, uni-professionally (99.26%). Concerning FHT, nurses were most dedicated to shared practices (33.09%) and exchanged information the most with others. According to team practices, the greatest amount of time dedicated to shared practices was spent by FHT (80%), followed by MultiT (71%) and OHT (65%).

**Conclusion:**

Multiprofessional work in PHC is evident as a precursor to interprofessional practice. In this sense, studies and reflections on the way in which PHC indicators have been monitored and evaluated, which mainly refer to individual activities, are required.

**Supplementary Information:**

The online version contains supplementary material available at 10.1186/s12913-025-12439-9.

## Introduction

Brazilian Primary Health Care (PHC) strengthening has taken place with the implementation of the Family Health Strategy (FHS). This comprises of a health care model consisting of, among various other features, teamwork-based service organization, exhibiting both advances and setbacks since its implementation [1, 2]

Communication is crucial in establishing achievement involvement, interaction and interdependence, taking place in regular meetings and gatherings, as well as in informal spaces, through different technologies. Communication also mediates activity sharing, although most research has focused on specific health workers, especially doctors. For example, some reviews have identified six types of interprofessional PHC collaborations, namely between doctors and nurses, PHC and specialized care doctors, doctors and pharmacists, doctors and mental health workers and intersectoral collaborations [3]

Work in daily health service routines is carried out essentially through collective activities, whether shared multi-professionally, involving different areas, or uni-professionally, involving workers belonging to the same professional area. The literature on the subject indicates interprofessional practice and education encouragement, with articulations between training systems and health work noted from the beginning of the 2000s, focusing on interprofessional collaborations due to user safety and health care quality contributions [4, 5]. Interprofessional PHC work, thus, aims to improve population health, patient experiences, the quality of life of health teams, care effectiveness and advancing in health equity, as well as health care quality [4–6].

Although emphasis is placed on the contributions education and interprofessional health practices bring to health systems, the literature points to terminological and conceptual inaccuracies as a barrier to the development and effective implementation of a consensus. This, in turn, makes it difficult to establish robust research, as clear term definitions are essential to good research practices [7, 8]. In this sense, Reeves, Xyrichis and Zwarenstein [7] proposed a distinction between four types of interprofessional practices, as follows: teamwork, collaboration, coordination and networking, anchored in the contingency approach. These distinctions allow for a greater understanding of healthcare team practices. Furthermore, conceptual interprofessionality structures are recognized as being built from various theoretical foundations associated with organizational theory, the sociology of professions, and critical theory, among others. Although a common perspective is observed, these concepts aim to overcome the traditional fragmented and medically centered health care model. Thus, actions must be guided by user and population health needs and seek greater integration, interaction and communication between agents [8]

The differences between interprofessional practices indicate that teamwork corresponds to a set of basic, but not restrictive, characteristics, namely shared identity, role clarity, interdependence, integration and co-responsibility [7]. In PHC, teams are responsible for the health conditions of both users and populations under their responsibility and, must, therefore, develop intense action integration and interdependence recognition to meet presented needs. Interprofessional practices also involve active user, family and community participation in decision-making processes regarding care plans and social control, in which action centrality must lie with health service users [9]

Interprofessional collaboration is required at all health levels, especially PHC, comprising a fundamental approach for comprehensive health care for users presenting multimorbidities, which is increasingly common in health systems, requiring that activities be shared by different professionals [7] Although studies have advanced in demonstrating the importance of interprofessional PHC practice, especially in the Brazilian model, with FHS no studies characterizing the time that workers dedicate to carrying out shared activities are available to date. This knowledge allows for the recognition of what and how much is carried out in terms of shared practice and can contribute to the advancement and strengthening of interprofessional practice in the Brazilian Unified Health System (*Sistema Único de Saúde*, SUS).

In this context, this study assessed shared practices in FHT, oral health teams (OHT) and multiprofessional teams (MultiT), in which health workers act together in PHC in the Brazilian context. This assessment advances knowledge by proposing shared activity analyses between different PHC team workers, as shared activities constitute an indication that these teams work together to meet user and population health needs. Shared work carried out in teams, comprises an alternative to the isolated and independent work of specific agents as a traditional health work model trait. The study therefore seeks to answer the question: What is the frequency of shared practice in the activities carried out by PHC team workers? To answer this question, the aim of the study was to identify the activities shared by PHC team workers, whether multiprofessional or uniprofessional.

## Methods

### Study location and data collection

This study was carried out in the city of São Paulo, SP, Brazil, in two southern region administrative districts, Campo Limpo and Vila Andrade. Together, these districts include 14 Basic Health Unit (BHU) units comprising about 1,032 active workers during the average collection period. Data were collected during two periods, the first from March to August 2021 and the second from March to August 2022, in five BHU selected for their convenience.

The coordinators of each BHU were contacted and following their consent, the study’s researchers held meetings to present the study aims and formalize health worker enrollment invitations. The ethical aspects of research were then contextualized and those who expressed interest and agreed to participate filled out a sociodemographic and work assessment questionnaire. The data collection period was then agreed upon and a schedule was defined with the BHU coordinators and health workers.

It is important to highlight that the data collection was carried out by a multiprofessional team of professionals hired specifically for this purpose (data collectors), consisting of dentists, nurses, and midwives. These professionals received comprehensive theoretical and practical training, totaling 160 hours, on the study's objectives and the data collection procedures, including instructions on how to carry out direct and continuous working hour observations of healthcare workers, ensuring consistency and rigor in the data acquisition process. Additionally, the team was supervised by two researchers to ensure that all steps were performed according to the protocols established in the research project.

The data collection process was conducted over a period of 179 days. The observation, without interaction or intervention, was carried out over five days, considering a typical work week for each professional. It began with the arrival of the healthcare professional at the BHU and concluded at the end of their shift, which varied between six to eight hours per day depending on the professional category being investigated. The data were recorded on a portable electronic device (tablet) used exclusively for this study and stored in the Research Electronic Data Capture [10, 11] data collection and management platform.

To guide this process, we used the instrument [12] designed to measure the workload of primary care health professionals, which was adapted for this study. The instrument included a list of direct care interventions (care provided directly to the user/family/community, such as a consultation) and indirect care interventions (care provided away from the user/family/community, but for their benefit, including actions aimed at managing the unit and care, such as administrative meetings), as well as related activities (activities that can be performed by other professionals from different categories), personal activities, waiting time, and absences. In addition, the instrument also allowed for the recording of the time and place where the professional carried out their activities, as well as indicating whether an intervention was performed in collaboration with another professional.

## Study design and inclusion criteria

This study comprises a descriptive observational assessment, employing the time-motion data collection methodology, comprising continuous and detailed observations carried out by an observer for each professional. In a Time-Motion study, which is a type of quantitative data gathering, an external observer records the actions and durations of each task performed by a healthcare professional during their work routine. This allows for an accurate capture of the time taken and the frequency with which workers dedicate themselves to each activity throughout a typical working day [13, 14]. Thus, external observers recorded all tasks performed by healthcare professionals and, in the present study, our analysis focused on shared activities that were carried out during the data collection period.

Regarding the inclusion criteria for enrollment, participants were required to be workers providing PHC services, with a minimum commitment of six months at the BHU and having been observed for at least one day during their working day. Activities in which the nature of the practice could not be clearly assessed were excluded from the study to ensure the consistency and reliability of the data collected. Furthermore, professionals who could not be observed throughout all activities of their daily routine were also excluded, as the lack of comprehensive observation could compromise the analysis of professional practices and their implications within the study context.

## Data analysis

**Fig 1:**
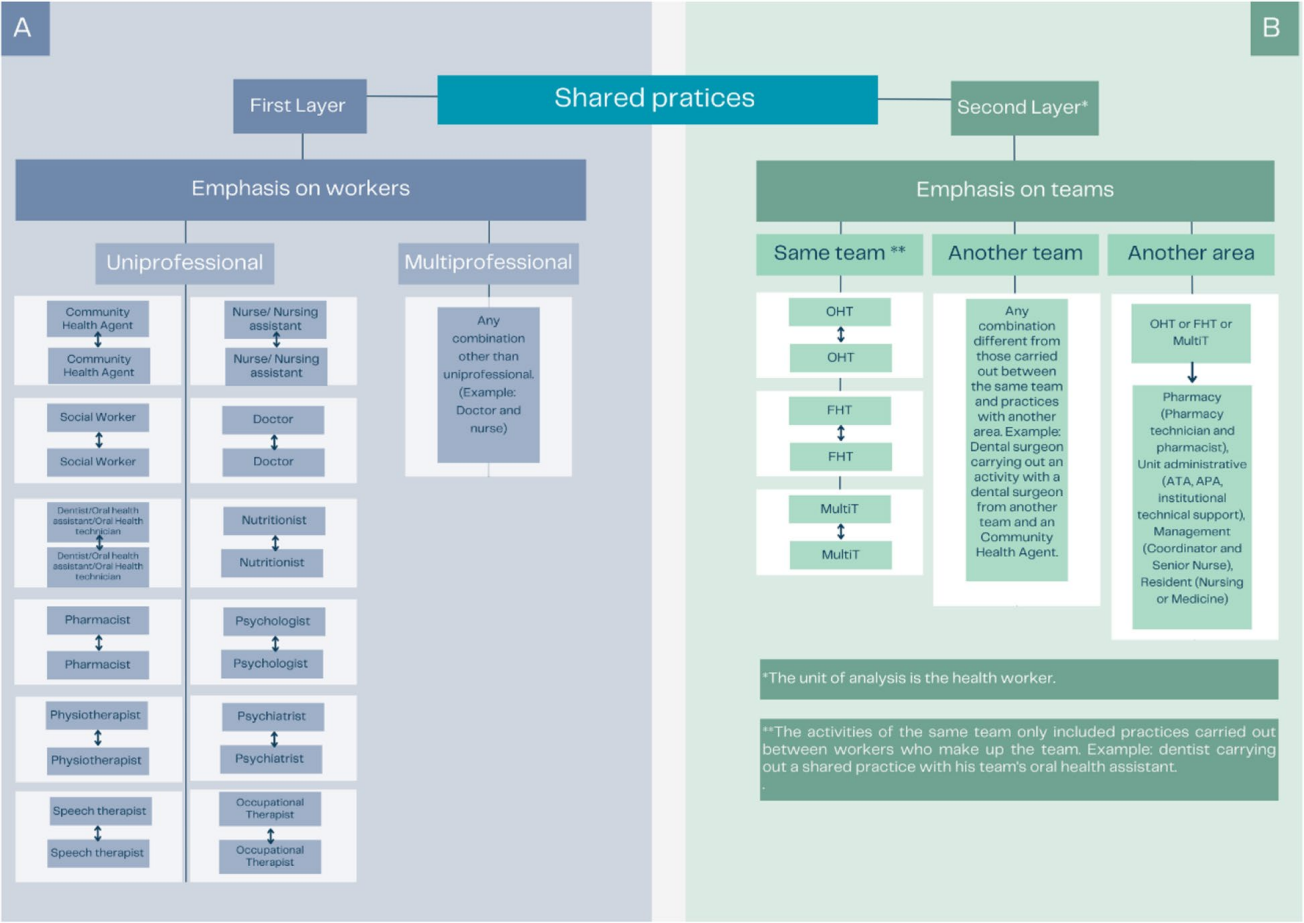
**a** and **b** Shared practices sub-classified and grouped according to professional area.

Workers in the same professional category were grouped together and recorded activities were classified as individual or shared practices, according to the presence or absence of other professionals during the observations. Activities were considered as shared if at least one worker other than the one being observed was involved. Additionally, the collection instrument favored the evaluation of shared practices for specific activities, by allowing the professionals to be identified (Supplementary Material 1). Shared practices were sub-classified and grouped according to professional area, resulting in two layers, one concerning workers and the other concerning teams (Figure 1a and b). Table 1 presents the estimate calculations, formula descriptions and illustrations of where they were used.

**Table 1:** Estimate calculations, formula description and illustrations where they were used

**Estimate**	**Description (Example)**	**Reference**
Activity Frequency rates	The number of observations of all workers in the same professional category was considered for each observed activity divided by the total number of activities observed for workers in the same professional category. **Example**: FRMC_I_ (Frequency rate of individual medical consultation) $${FRMC}_{I}=\left(\frac{Number of Medical Consultations observed individually}{Total medical consultations observed}\right)x 100$$	Supplementary Table 1
Activity time rates	The time observed for each activity of all workers in the same professional category was summed and divided by the total time recorded for each activity among workers in the same professional category. **Example**: SMCTR_C_ (Shared medical consultation time rate) $${SMCTR}_{C}=\left(\frac{Sum of time for shared medical appointments}{Sum time of the total medical consultations observed}\right)x 100$$	Supplementary Table 11
Uni-professional and multi-professional shared practice time rates	Using the groupings depicted in Figure 1a and 1b, the time recorded for uni-professional and multiprofessional shared activities was grouped for each type of shared activity according to professional category. The rate followed the same logic as the time rate. **Example**: DUSPTR_UM_ (Doctor's uniprofessional shared practice time rate) $${DUSPTR}_{um}=\left(\frac{Time spent on uniprofessional practice activities of the physician}{Total time of the physician{\prime}s uniprofessional practices}\right)x 100$$	Figure 2
Time rates between teams	The time rates for the teams' shared practice activities were classified into four categories, Figure 1b, and calculated following the same logic as the activity time rates. **Example**: PTRS_OE_ of FHT (Practice time rate shared with other teams in the FHT) $${PTRS}_{OE} of FHT=\left(\frac{Time for shared practice activities of FHTworkers with other teams}{Total time of shared practice activities of FHT workers)}\right)x 100$$	Figure 3
Rate of professionals involved in information exchange	Exchanges of information involving only one professional were considered for this calculation; those that included more than one professional were excluded from this sub-analysis. The rates follow the same method as the frequency rate.	Table 1

**Fig 2:**
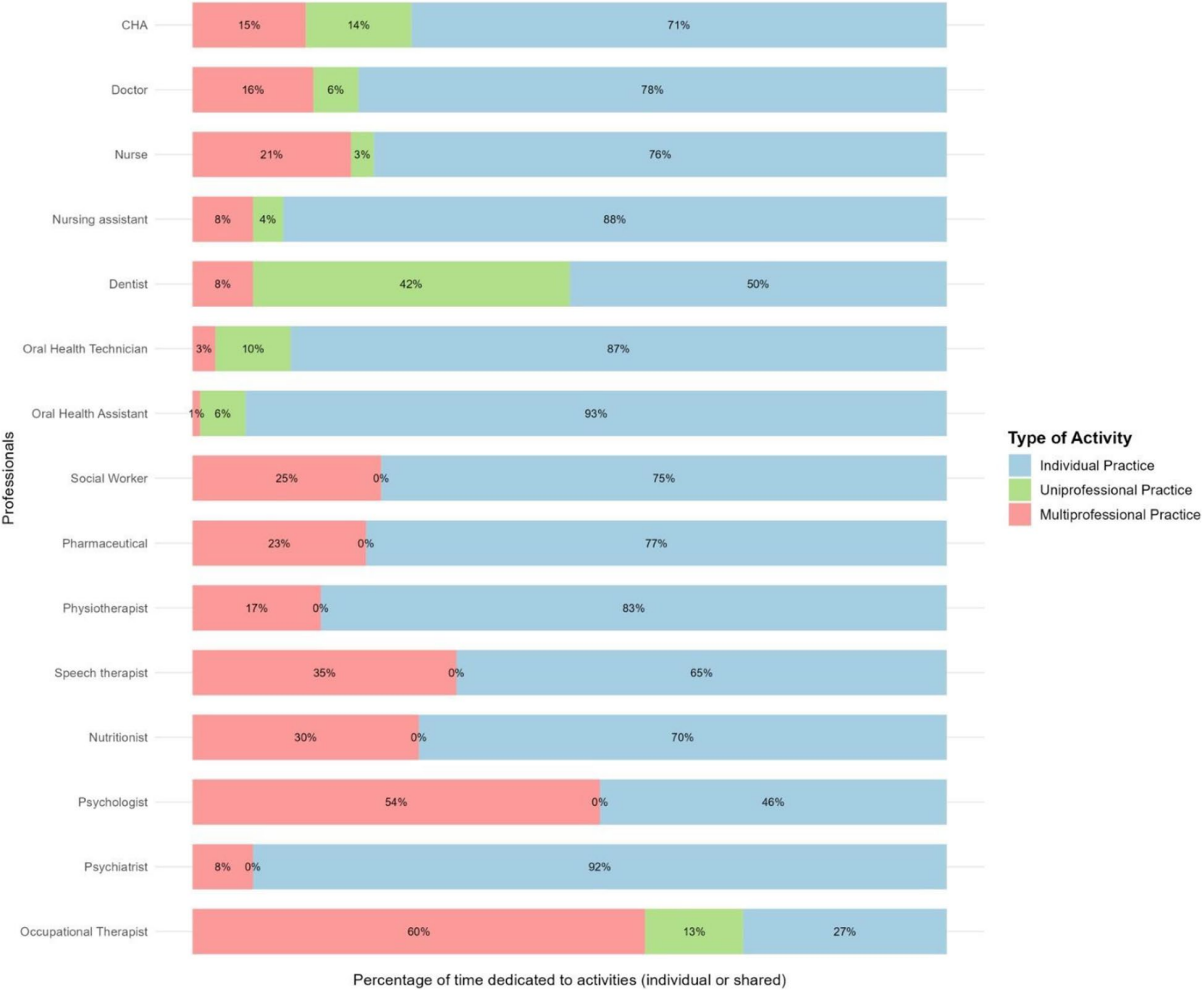
Time dedicated to shared practices according to each evaluated category

**Fig 3:**
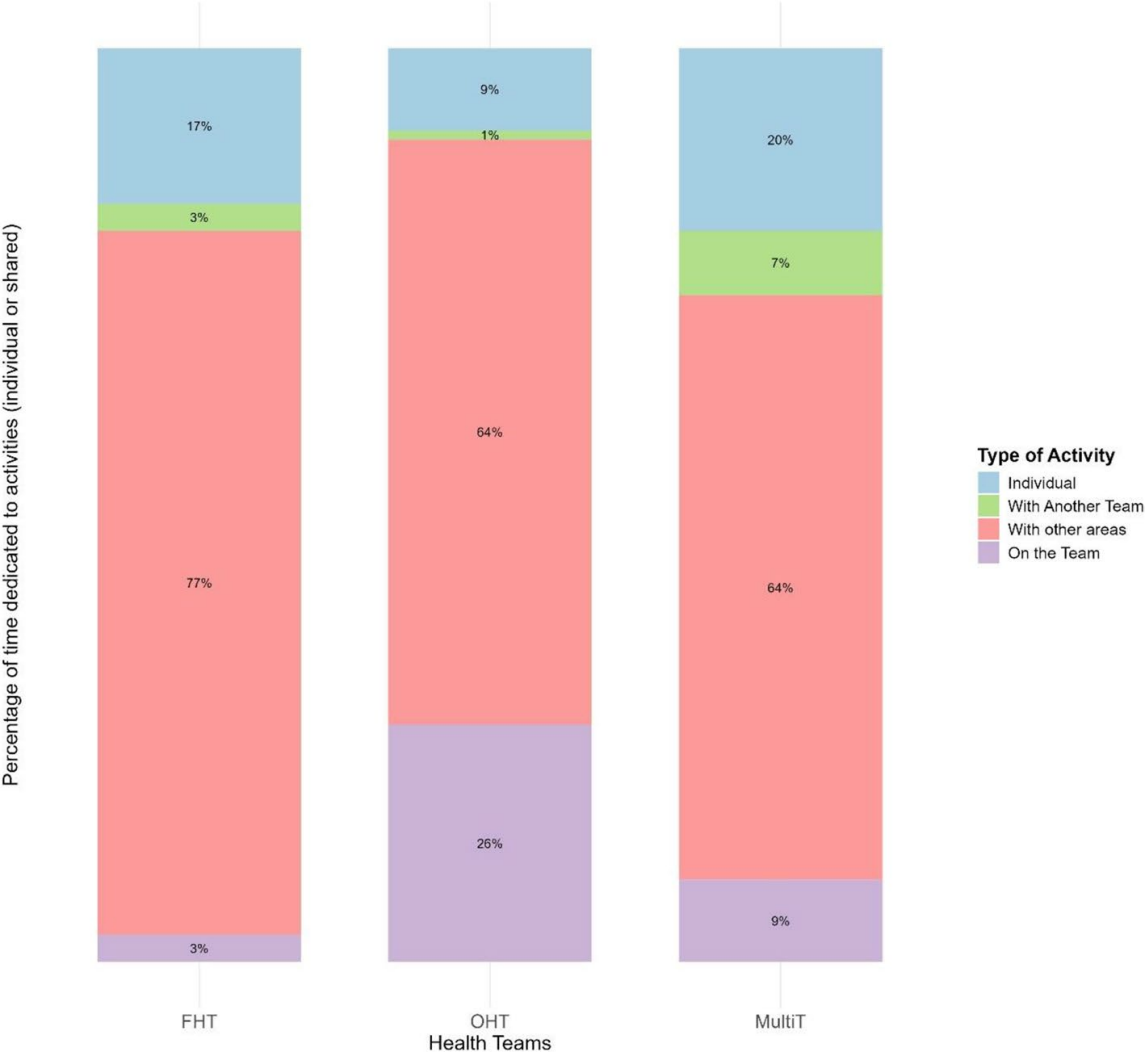
Time distributions according to the type of practice by the investigated PHC team

The applied analysis unit comprised individual or shared activities carried out by workers. Shared practices were categorized as uniprofessional when exchanges were carried out by the same professional category, such as between doctors, between nurses and nursing assistants; between dentists and oral health technicians (OHTec) or oral health assistants (OHA), among others. The second type, multiprofessional, considered exchanges between workers belonging to different categories, such as between nurses and doctors and between pharmacists and nurses, among others. The analyses were performed using Excel software.

### Ethical aspects

This study is associated to a larger survey entitled “Patient Panel for Teams in the Family Health Strategy”, approved by the research ethics committee of Albert Einstein Israelita Hospital (n. 4.746.712; CAAE: 23388819.9.0000.0071) and by the São Paulo Municipal Health Department (n 3.794.021; CAAE: 23388819.9.3001.0086). Workers were invited to enroll during the research presentation at each BHU, and those favorable were instructed to access an electronic form using a QR code to read and sign the Free and Informed Consent Form, if accepting enrollment.

## Results

Of the total number of Primary Health Care (PHC) workers evaluated in this study (*n*=93), the majority were female (88.17%), with a mean age of 36.25 years. The distribution of the workers was as follows: 22.58% (*n*=21) were nurses, 19.35% (*n*=18) community health agents (CHA), 16.13% (*n*=15) nursing assistants, 12.90% (*n*=12) dentists, 10.75% (*n*=10) doctors, 8.60% (*n*=8) multiprofessional workers (psychologists, nutritionists, social workers, speech therapists, psychiatrists, and occupational therapists), 5.38% (*n*=5) oral health technicians (OHT), 3.23% (*n*=3) oral health assistants (OHA), and 1.07% (*n*=1) were pharmacists. Among the 89 workers who provided complete sociodemographic and professional information, 32.58% reported having at least one postgraduate degree, such as a specialization, MBA, master's, or doctorate.

The sample consisted of a total of 21,932 observed activities for all monitored workers, totaling 157,631 minutes. Of these, 73.76% (116,263 minutes) corresponded to individual activities and 26.24% (41,368 minutes) to shared activities (41.91% uniprofessional and 58.09% multiprofessional). Of the accounted individual activities (73.76%) of the total observed time, with notable activities where most of the observed time was individual: immunization/vaccine control (100%), documentation (99.92%), work process organization (99.56%), associated activities (99.01%), consultations (93.35%), and home visits (55.78%).

Shared activities accounted for 41,368 minutes, equivalent to 26.24% of the observed time of the workers included in the study. Of these, multiprofessional shared practices represented 15.24% of the total time, and uniprofessional shared practices represented 11.00% of the total time.

Regarding multiprofessional shared practices, it is noteworthy that 71.82% of the exchanges of information about health care and/or health services for users occurred in a multiprofessional basis. Additionally, meetings are frequently multiprofessional in nature, with worker participation observed in a total of 113 administrative meetings, the majority of which were multiprofessional (71.68%), involving up to 15 workers, with a total time of 6,446 minutes. Administrative meetings represented 2.81% of the workers' observed time. The worker categories with the most time dedicated to administrative meetings were dentists, doctors, and nurses.

Regarding multiprofessional meetings, 86 meetings were observed with a total time of 4,427 minutes. These meetings included matrix support with the MultiT, with the Psychosocial Care Centers (CAPS) for alcohol and drugs, adults, and children, team meetings, meetings of the violence prevention core, among others. Information on the time dedicated to shared and individual practices according to each evaluated category is presented in Figure 2. FHS workers predominantly dedicate their time to individual activities, with doctors spending the most time on individual practices (78.00%), followed by nurses (76.00%) and CHA (71.00%). Among these categories, nurses dedicated the most time to working with other professionals, spending 21.00% of their time on multiprofessional shared activities. As for dentists, 42.00% of their time is dedicated to uniprofessional sharing with workers in the oral health field. Regarding the MultiT, these teams had less observed time and fewer workers, with occupational therapists (73.00%), psychologists (54.00%), speech therapists (35.00%), and psychiatrists (8.00%) spending more time on shared practices. Uniprofessional shared practices were observed during the MultiT observations, as this team is multiprofessional by nature.

From the perspective of shared activities, home visits stood out for the CHA, representing 53.68% of the total observed time for this category. For nurses, doctors, and dentists, consultations stood out with 15.16%, 6.38%, and 68.07% of the observed time, respectively. The detailed results of these activities, considering time and number of activities, are presented in Supplementary Material 2.

Specifically for the dentist, through the registered four-handed consultations, it was possible to highlight that these were mainly performed in a uniprofessional shared manner (99.6%). Although Figure 2 shows that shared activity in the OHT occurs mostly by the dentist, it is noted that for the OHTec and OHA, it was not recorded with whom these workers interacted in support activities for procedures or exams performed by these two categories; thus, the percentage of shared practices among technicians/assistants may not be reflected in the calculations presented.

Among the activities, some are exclusively characterized as shared activities, such as the exchange of information about health care and/or health services. The time spent on this activity by nurses was 13.37%, for doctors 8.44%, and for dentists 6.39%. The workers who exchanged the most information with other categories were nurses, collaborating with doctors (34.70%), nursing assistants (25.80%), other nurses (25.50%), and CHA (10.30%). Information regarding the exchange of information about health care and/or health services performed by each worker category is presented in Tables 2 and 3.

**Table 2:** Worker rate distribution involved in information exchanges carried out by FHT workers

**Category**	**Doctor**		**Nurse**		**Nursing assistant**		**Dentist**		**Oral Health Assistant**		**Oral Health Technician**		**Community Health Agent**	
	**N**	**%**	**N**	**N**	**%**	**%**	**N**	**%**	**N**	**%**	**N**	**%**	**N**	**%**
**Doctor**	101	22.5	514	11	3.7	15.4	11	3.7	0	0.0	3	1.9	41	12.9
**Nurse**	235	52.3	377	27	9.0	26.4	27	9.0	0	0.0	5	3.1	74	23.3
**Nursing assistant**	45	10.0	382	12	4.0	44.4	12	4.0	3	4.5	5	3.1	35	11.0
**Dentist**	1	0.2	10	58	19.3	0.9	58	19.3	38	57.6	88	55.3	14	4.4
**Oral Health Assistant**	0	0.0	1	72	24.0	2.8	72	24.0	12	18.2	29	18.2	7	2.2
**Oral Health Technician**	2	0.4	5	95	31.7	0.9	95	31.7	9	13.6	24	15.1	0	0.0
**Community Health Agent**	51	11.4	152	14	4.7	6.9	14	4.7	2	3.0	3	1.9	133	42.0
**Nutritionist**	3	0.7	3	0	0.0	0.7	0	0.0	0	0.0	0	0.0	5	1.6
**Pharmacist**	3	0.7	9	3	1.0	0.2	3	1.0	1	1.5	0	0.0	1	0.3
**Pharmacy Technician**	1	0.2	15	5	1.7	1.1	5	1.7	1	1.5	1	0.6	3	0.9
**Physiotherapist**	1	0.2	0	0	0.0	0.0	0	0.0	0	0.0	0	0.0	0	0.0
**Psychologist**	1	0.2	3	3	1.0	0.2	3	1.0	0	0.0	0	0.0	1	0.3
**Psychiatrist**	2	0.4	1	0	0.0	0.0	0	0.0	0	0.0	1	0.6	1	0.3
**Occupational Therapist**	0	0.0	1	0	0.0	0.0	0	0.0	0	0.0	0	0.0	0	0.0
**Speech therapist**	1	0.2	6	0	0.0	0.0	0	0.0	0	0.0	0	0.0	1	0.3
**Social Worker**	2	0.4	1	0	0.0	0.0	0	0.0	0	0.0	0	0.0	1	0.3
**Total**	**449**	**100**	**1480**	**300**	**100**	**100**	**300**	**100**	**66**	**100**	**159**	**100**	**317**	**100**

**Table 3:** Worker rate distribution involved in information exchanges carried out by MultiT workers

**Category**	**Psychologist**		**Psychiatrist**		**Nutritionist**		**Occupational Therapist**		**Social Worker**		**Pharmacist**		**Physiotherapist**		**Speech therapist**	
	**N**	**%**	**N**	**%**	**N**	**%**	**N**	**%**	**N**	**%**	**N**	**%**	**N**	**%**	**N**	**%**
**Doctor**	7	12.5	7	12.5	3	8.3	0	0.0	0	0.0	5	21.7	6	26.1	5	15.6
**Nurse**	16	28.6	16	28.6	7	19.4	0	0.0	7	36.8	4	17.4	7	30.4	9	28.1
**Nursing assistant**	2	3.6	2	3.6	0	0.0	0	0.0	0	0.0	1	4.3	2	8.7	0	0.0
**Dentist**	0	0.0	0	0.0	0	0.0	0	0.0	0	0.0	0	0.0	0	0.0	0	0.0
**Oral Health Assistant**	0	0.0	0	0.0	0	0.0	0	0.0	0	0.0	0	0.0	0	0.0	0	0.0
**Oral Health Technician**	0	0.0	0	0.0	0	0.0	0	0.0	0	0.0	0	0.0	0	0.0	0	0.0
**Community Health Agent**	5	8.9	5	8.9	3	8.3	1	100	3	15.8	1	4.3	8	34.8	6	18.8
**Nutritionist**	11	19.6	11	19.6	0	0.0	0	0.0	0	0.0	0	0.0	0	0.0	12	37.5
**Pharmacist**	0	0.0	0	0.0	2	5.6	0	0.0	0	0.0	1	4.3	0	0.0	0	0.0
**Pharmacy Technician**	0	0.0	0	0.0	0	0.0	0	0.0	0	0.0	11	47.8	0	0.0	0	0.0
**Physiotherapist**	8	14.3	8	14.3	6	16.7	0	0.0	0	0.0	0	0.0	0	0.0	0	0.0
**Psychologist**	2	3.6	2	3.6	11	30.6	0	0.0	0	0.0	0	0.0	0	0.0	0	0.0
**Psychiatrist**	1	1.8	1	1.8	1	2.8	0	0.0	1	5.3	0	0.0	0	0.0	0	0.0
**Occupational Therapist**	1	1.8	1	1.8	1	2.8	0	0.0	1	5.3	0	0.0	0	0.0	0	0.0
**Speech therapist**	2	3.6	2	3.6	2	5.6	0	0.0	7	36.8	0	0.0	0	0.0	0	0.0
**Social Worker**	1	1.8	1	1.8	0	0.0	0	0.0	0	0.0	0	0.0	0	0.0	0	0.0
**Total**	**56**	**100**	**56**	**100**	**36**	**100**	**1**	**100**	**19**	**100**	**23**	**100**	**23**	**100**	**32**	**100**

The locations where workers shared activities included, mostly, offices (34.08%), dental offices (16.68%), and hallways of the BHU (7.98%). Nurses were the most mobile between sectors for sharing activities (39.70%). Regarding the sharing locations by team, the majority for the FHT occurred in the office, for the OHT in the dental office, and for the MultiT in the room designated for that team.

The figure 3 presents the time distributions according to the type of practice by the investigated PHC teams, with the longest shared activities with other teams and areas performed by the FHT (80%), followed by MultiT (71%) and OHT (65%)

## Discussion

The findings reported herein indicate that PHC activities consist of both individual and shared practices by workers and worker teams, with different times dedicated to different activities. About one quarter of the activities observed among FHS team workers were carried out in a shared manner, mostly multiprofessional and not uniprofessionally. The rest of the activities were carried out individually by each team worker, like the team-based work organization model recommended by the FHS in SUS PHC.

In this sense, PHC organization seeks to overcome fragmented and isolated care for each profession according to the biomedical model, which comprises a service production centered on disease and uniprofessional work that tends to mechanize health care and care technification [15]. In relation to shared activities, studies indicate that effective multiprofessional teamwork has the power to advance the integration of activities, interaction and communication between workers and teams, as well as interprofessional collaboration activities that, according to the adopted framework, correspond to interprofessional work [7, 8, 16].

It is well known that interprofessional care in PHC plays a crucial role in the comprehensive care of users, and despite the expansion of interprofessional teams in this scenario, some professions are still underrepresented despite being involved in providing care and often act in an orbital way to the FHT [17]. The sporadic involvement of the professionals from MultiT shows that interprofessional practice still needs to advance, although the data from this research suggests progress in that direction. It should be noted that in the Brazilian model of FHS, users and families are assigned to FHT and OHT, but the support of MultiT is done through periodical visits to the BHU.

Most of the observed administrative meetings were multiprofessional, with the presence of workers belonging to different categories and areas. The recorded multiprofessional meetings included various activities, such as matrix support with MultiT and CAPS teams and team meetings, among others. The exchange of user care information also comprised multiprofessional shared activities. Both formal and informal exchange spaces contribute to strengthening teamwork and collaborative practices [8, 16, 18] comprising offices and BHU corridors in the present study. One study carried out in a hospital environment also highlighted informal exchanges as part of collaborative worker practices [19]. In addition to promoting interpersonal relationships, respect and trust, informal exchanges also allow for quick problem-solving and decision-making [20]

Spaces for worker and team exchanges reflect the PHC organization and work management model. This was noted in another a study that identified aspects associated to organizational collaboration influence, focusing on vision and objective clarity, strategic coordination and communication mechanisms between the workers themselves and between workers and the served users and population [21]. The literature on interprofessional work points to communication as a key element, both in the four modalities determined by Reeves [7] (teamwork, collaborative practice, coordination and networking) and concerning collaborative skills, which are considered the core competence of collaborative practice [9, 16, 22, 23]

The findings obtained herein also indicate that nurses dedicated more time to shared activities, in particular consultations and information exchange. This corroborates other assessments that highlight the role that nurses play in sharing activities with other PHC workers, comprising a link between agents, receiving centers and information disseminators, as well as important in the articulation of individual health worker activities [24, 25]

In relation to the factors associated with interprofessional teamwork in primary care settings, a Canadian study concluded that the adoption of various communication mechanisms to share information, improved quality and practice time had a statistically significant positive association; on the other hand, increased team size and the use of centralized administrative processes were negatively associated with the Collaborative Practice Assessment Tool score, demonstrating that the factors that enable interprofessional teamwork in primary care teams is still in the begging [26].

Regarding OHT care sharing, most exchanges took place between dentists, OHTec and OHA, mainly during individual dental procedure consultations, although coordinated care practices with other health teams are within the scope of these workers. In a Covid-19 pandemic scenario, OHT needed to reorganize their work process, as did other teams [27]. Concerning OHT, elective services were suspended, emergencies were prioritized and teledentistry was introduced. In some studies, OHT resistance was observed in following new recommendations to adapt care services, with attention mostly focused on clinical and individual interventions during the pandemic [28] and difficulty of these teams in acting interprofessionally [29]. In this scenario, an increase in the interprofessional collaborative spirit was identified in various countries worldwide, including Brazil, where space to incorporate more in-depth debates concerning interprofessional health teams were noted [28]. Interprofessional collaborative practices are, in fact, one of the sustainable ways to deal with emergency situations and require collaborative high-quality and safe efforts focusing on users [30, 31]

MultiT in Brazil originate from Expanded Family Health Centers (equipes multiprofissionais na atenção primária - eMulti), and their activities in BHU include both clinical assistance activities and pedagogical techniques in the form of matrix support, with the aim of producing joint actions with user reference teams based on collaborative work [32]. In this study, the time dedicated by MultiT professionals in activities carried out with other teams and other areas stands out, reinforcing what national policy foresees for these teams. However, as for the other teams, the quality of the exchange interactions was noted assessed, although a proposal to evaluate the effectiveness of MultiT, through a judgment matrix, constructed from the fundamental concepts that guide the work process of this important Brazilian PHC device, which includes teamwork, has been noted in one assessment [33]

We reinforce that OHT is the priority strategy of the health care network, with support from MultiT [34] as increasing chronic diseases, population aging and health complexity require PHC to provide comprehensive, continuous and coordinated care provided by several health workers, organized in teams and in networks. Although this study did not analyze collaboration levels and interaction quality between workers, shared activities are indicative of teamwork and precursors to interprofessional collaboration [5, 35]

Concerning shared activities, ranging from occasional information exchanges to user care consultations, the latter expands the possibility of collective decision-making and inter-worker collaboration. It is important to note that the conceptual framework for interprofessional collaborative practice focuses on four core competencies, namely values ​​and ethics, maintaining a climate of shared values, ethical conduct and mutual respect; roles and responsibilities, using the knowledge and skills of one's own role and that of others to achieve health results for users and populations; communication in an agile, responsible, respectful and compassionate way; teams and teamwork, applying values ​​and principles for teamwork to adapt their own role in diverse environments [23] which are only achieved by sharing daily activities, the object studied herein.

Although the results reported herein demonstrate progress concerning the team-based PHC organization model, focusing on shared activities, multiprofessional work has experienced adversities since the mid-2010s, with threats noted to the current care model, especially due to the financing logic of the Previne Brasil 2019 Program. In this program, the transfer of resources is based on biological and procedural result indicators, which can reinforce isolated worker practices, mainly doctors and nurses, as noted for the primary activities carried out by these professionals [36, 37] The Previne Brasil Program was in place during the data collection period, however, more recently, there has been a change in the federal co-financing model for the PHC within the Brazilian Unified Health System (SUS) through the ordinance 3.493/2024, [38] aims to encourage continuous improvement in the quality of primary care, providing a more responsive health system.

It is important that future studies investigate beyond individual and shared uni- and multi-professional activities and time frames, also evaluating these under inter-professional perspectives, which require a qualitative approach or the application of mixed methods to evaluate interaction quality. Thus, the findings from this research indicate the need for further studies, particularly from a qualitative perspective, to evaluate the quality and effectiveness of the shared activities.

Some limitations are noted for this study. First, when defining the worker groupings, a lack of weighting of team rates was noted based on the way the data was collected and the applied instrument, as activities carried out by workers with higher education were prevalent compared to activities carried out by secondary level workers. The field research method (time-motion) and the employed database also do not allow for assessments concerning the quality of shared activities, as well as their objectives, resolvability and integration. Concerning the analyses, we assume that, when calculating dispersion and distribution measures, each observed activity is unique and independent of professional category and individual characteristics. Additionally, the number of follow-up observations (time and days), which are different for each professional category and within categories, could not be assessed.

## Conclusions

The results of the study show the importance of shared activities in the context of Brazilian PHC, accounting for around a quarter of all activities carried out, reaffirming that the PHC organization model seeks to overcome fragmented and isolated care by each profession.

Among team practices, the most time dedicated to shared practices was spent by the FHT, followed by MultiT and OHT. The shared activities identified were multiprofessionals meetings, consultations, exchanges of information and home visits, with a variation in the time observed for each professional area. Nurses spent the most time on consultation and information exchange activities and were also the professionals who physically moved around the unit the most in search of sharing.

Another highlight is the professionals who make up the MultiT, who, despite having less time and fewer professionals, dedicated more time to shared practices, indicating that these teams are fundamental for collaborative work in PHC. OHT professionals, on the other hand, tended to carry out shared activities on a uniprofessional basis among the professionals in the area, such as dentists, OHTec, and OHA, due to the characteristics of the work they do, and less so with other professionals from FHT and MultiT.

The shared activities observed constitute a precursor to interprofessional work, as the type of activity and the time dedicated to its execution revealed that workers collaborate to integrate their knowledge and practices with the knowledge of users and the population in addressing health needs.

In this sense, studies and reflections on the way in which PHC indicators are being monitored and evaluated are required which refer mainly to activities carried out individually by each worker, such as consultation and technical procedures, as well as the collection of production targets by these workers and not by teams. The findings reported herein contribute preliminarily to the development of interprofessional work indicators, highlighting a significant set of shared user and population care activities.

## Supplementary Information

Supplementary Material 1.

Supplementary Material 2.

## Data Availability

The data underlying this study contains potentially sensitive information and sharing them openly could compromise the privacy and confidentiality of the study participants. In accordance with ethical guidelines and to protect participant confidentiality, the raw data cannot be made publicly available. However, interested researchers may request access to a de-identified and aggregated dataset by contacting the authors. Requests will be considered on a case-by-case basis, and approval will be contingent upon ensuring the continued protection of participant privacy.

## Abbreviations

BHU: Basic Health Unit
*Sistema Único de Saúde* – SUS: Brazilian Unified Health System
CHA: Community Health Agents
DUSPTR_UM_: Doctor's uniprofessional shared practice time rate
FHS: Family Health Strategy
FHT: Family Health Teams
FRMC_I_: Frequency rate of individual medical consultation
MultiT: Multiprofessional teams
OHA: Oral Health Assistants
OHT: Oral Health Teams
OHTec: Oral Health Technicians
PHC: Primary Health Care
*Centro de Atenção Psicossocial* – CAPS: Psychosocial Care Centers
PTRS_OE_: Practice time rate shared with other teams
SMCTR_C_: Shared medical consultation time rate
