# Supplementary figures and images for: Shared practices among primary health care workers: A time-motion study
Source: BMC Health Serv Res. 2025 Feb 26;25:317. doi: 10.1186/s12913-025-12439-9 (PMC11863654; doi:10.1186/s12913-025-12439-9)

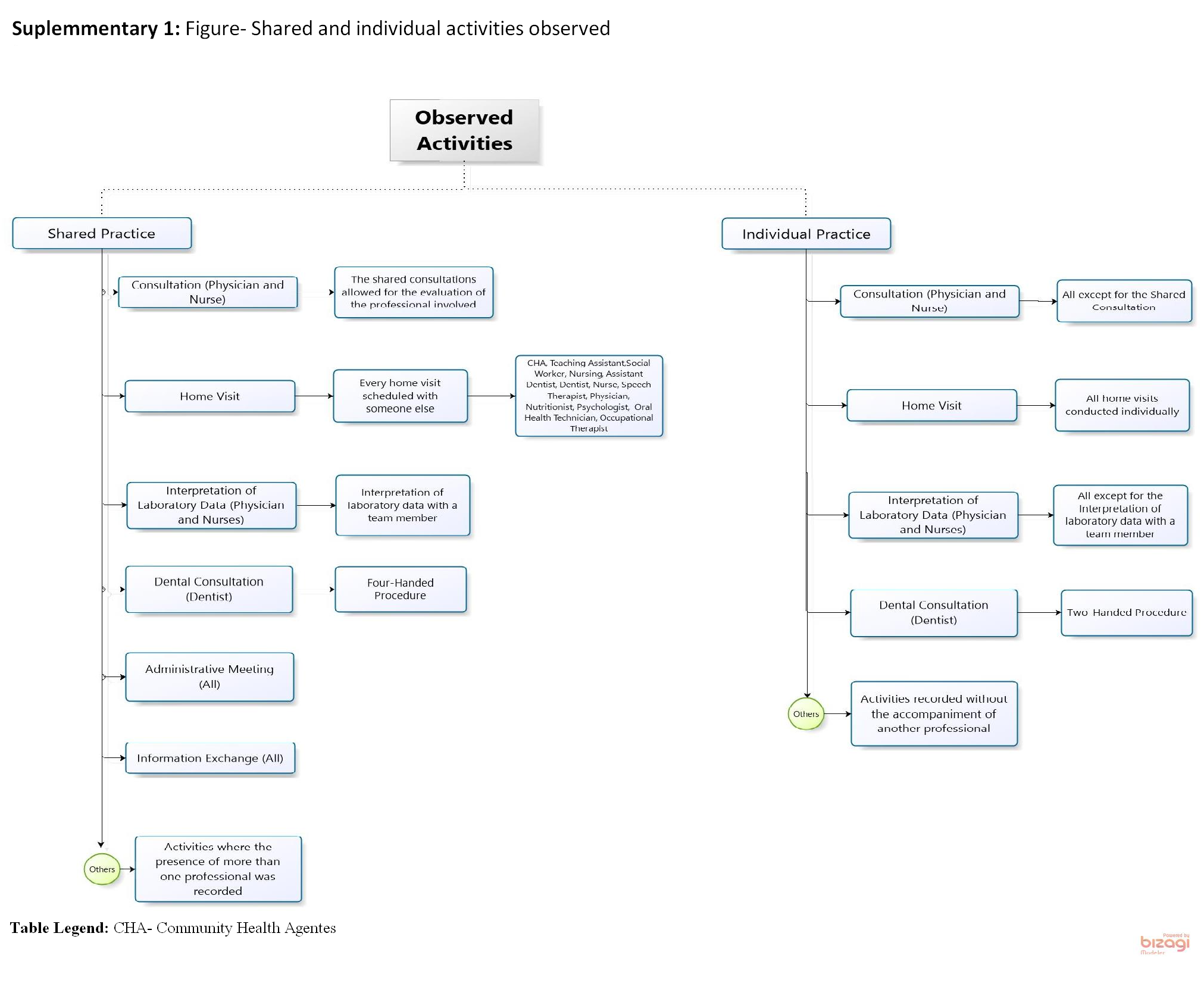

Supplement: Supplementary file 1 — Supplementary Material 1. [file 12913_2025_12439_MOESM1_ESM.png]
