# Supplementary material for: Shared practices among primary health care workers: A time-motion study
Source: BMC Health Serv Res. 2025 Feb 26;25:317. doi: 10.1186/s12913-025-12439-9 (PMC11863654; doi:10.1186/s12913-025-12439-9)
Supplement: Supplementary file 2 — Supplementary Material 2. [file 12913_2025_12439_MOESM2_ESM.docx]

**Suplemmentary 2:** Table- Rate of individual and shared activities by professional category

| **Professional category** | **Acommunity health agent** | | | | | | | | | | | | | | | | | |
| --- | --- | --- | --- | --- | --- | --- | --- | --- | --- | --- | --- | --- | --- | --- | --- | --- | --- | --- |
|  | **Shared practice** | | | | | | **Individual practice** | | | | | | |  | | | | |
| **Activities** | **TR*** | | **TR**** | | | **TR*** | | | | **TR**** | | | **Total*** | | | **Total**** | | |
| Educational actions for health care professional (permanent training) | 0 | | 0 | | | 100 | | | | 100 | | | 4 | | | 257 | | |
| Infection control | 0 | | 0 | | | 100 | | | | 100 | | | 5 | | | 12 | | |
| Documentation | 0,38 | | 0,29 | | | 99,61 | | | | 99,70 | | | 523 | | | 10147 | | |
| Risk identification | 0 | | 0 | | | 100 | | | | 100 | | | 15 | | | 200 | | |
| Mapping and territorialization | 0 | | 0 | | | 100 | | | | 100 | | | 1 | | | 31 | | |
| Organization of the work process | 0 | | 0 | | | 100 | | | | 100 | | | 326 | | | 1806 | | |
| Advice on the Health Care Sytem | 0,52 | | 0,72 | | | 99,47 | | | | 99,27 | | | 384 | | | 963 | | |
| Promotion of education activities | 0 | | 0 | | | 100 | | | | 100 | | | 1 | | | 47 | | |
| Reference and cross-reference | 0 | | 0 | | | 100 | | | | 100 | | | 1 | | | 2 | | |
| Administrative meetings | 100 | | 100 | | | 0 | | | | 0 | | | 18 | | | 605 | | |
| Multidisciplinary care assessment meetings | 100 | | 100 | | | 0 | | | | 0 | | | 29 | | | 1521 | | |
| Safety monitoring | 0 | | 0 | | | 100 | | | | 100 | | | 3 | | | 61 | | |
| Exchange of information about health care and/or health services | 100 | | 100 | | | 0 | | | | 0 | | | 429 | | | 1338 | | |
| Health surveillance | 0 | | 0 | | | 100 | | | | 100 | | | 3 | | | 42 | | |
| Home visits | 38,47 | | 53,68 | | | 61,52 | | | | 46,31 | | | 1380 | | | 9025 | | |
| Associated activities | 0,93 | | 1,14 | | | 99,06 | | | | 98,85 | | | 859 | | | 2878 | | |
| Performance evolution | 0 | | 0 | | | 100 | | | | 100 | | | 1 | | | 11 | | |
| Immunization control/Vaccionation | 0 | | 0 | | | 100 | | | | 100 | | | 31 | | | 55 | | |
| **Rate for all activities with both modalities** | **25,39** | | **28,89** | | | **74,60** | | | | **71,10** | | | **4013** | | | **29001** | | |
| **Rate of the main activity** | **38,47** | | **53,68** | | |  | | | |  | | |  | | |  | | |
| **Professional category** | **Social Worker** | | | | | | | | | | | | | | | | | |
|  | **Shared practice** | | | **Individual practice** | | | | | | | |  | | | | | | |
| **Activities** | **TR*** | **TR**** | | | **TR*** | | | | **TR**** | | | **Total*** | | | **Total**** | |  |  |
| Infection control | 0 | 0 | | | 100 | | | | 100 | | | 3 | | | 3 | |  |  |
| Documentation | 0 | 0 | | | 100 | | | | 100 | | | 17 | | | 262 | |  |  |
| Organization of the work process | 0 | 0 | | | 100 | | | | 100 | | | 26 | | | 276 | |  |  |
| Advice on the Health Care Sytem | 0 | 0 | | | 100 | | | | 100 | | | 5 | | | 28 | |  |  |
| Administrative meetings | 100 | 100 | | | 0 | | | | 0 | | | 1 | | | 96 | |  |  |
| Exchange of information about health care and/or health services | 100 | 100 | | | 0 | | | | 0 | | | 29 | | | 126 | |  |  |
| Home visits | 100 | 100 | | | 0 | | | | 0 | | | 5 | | | 84 | |  |  |
| Associated activities | 0 | 0 | | | 100 | | | | 100 | | | 14 | | | 38 | |  |  |
| Consutation | 0 | 0 | | | 100 | | | | 100 | | | 11 | | | 302 | |  |  |
| **Rate for all activities with both modalities** | **31,53** | **25,18** | | | **68,46** | | | | **74,81** | | | **111** | | | **1215** | |  |  |
| **Rate of the main activity** | **0** | **0** | | |  | | | |  | | |  | | |  | |  |  |
| **Professional category** | **Dentist** | | | | | | | | | | | | | | | |  |  |
|  | **Activities** | | | | | | **Individual practice** | | | | |  | | | | |  |  |
| **Activities** | **TR*** | **TR**** | | | **TR*** | | | | **TR**** | | | **Total*** | | | **Total**** | |  |  |
| Educational actions for health care professionals (master’s/doctoral courses) | 0 | 0 | | | 100 | | | | 100 | | | 2 | | | 206 | |  |  |
| Educational actions for health care professional (permanent training) | 0 | 0 | | | 100 | | | | 100 | | | 4 | | | 330 | |  |  |
| Infection control | 0 | 0 | | | 100 | | | | 100 | | | 100 | | | 148 | |  |  |
| Supply control | 0 | 0 | | | 100 | | | | 100 | | | 3 | | | 14 | |  |  |
|  |  |  | | |  | | | |  | | |  | | |  | |  |  |
| Community health development | 0 | 0 | | | 100 | | | | 100 | | | 1 | | | 2 | |  |  |
| Development of care processes and | 0 | 0 | | | 100 | | | | 100 | | | 4 | | | 239 | |  |  |
| Documentation | 0 | 0 | | | 100 | | | | 100 | | | 327 | | | 3107 | |  |  |
| Mapping and territorialization | 0 | 0 | | | 100 | | | | 100 | | | 1 | | | 11 | |  |  |
| Organization of the work process | 0,46 | 0,18 | | | 99,53 | | | | 99,81 | | | 216 | | | 1594 | |  |  |
| Advice on the Health Care Sytem | 0 | 0 | | | 100 | | | | 100 | | | 40 | | | 108 | |  |  |
| Outpatient Procedures | 0 | 0 | | | 100 | | | | 100 | | | 8 | | | 99 | |  |  |
| Collective procedures | 0 | 0 | | | 100 | | | | 100 | | | 18 | | | 188 | |  |  |
| Promotion of education activities | 0 | 0 | | | 100 | | | | 100 | | | 1 | | | 9 | |  |  |
| Reference and cross-reference | 0 | 0 | | | 100 | | | | 100 | | | 1 | | | 11 | |  |  |
| Administrative meetings | 100 | 100 | | | 0 | | | | 0 | | | 25 | | | 1502 | |  |  |
| Student support | 0 | 0 | | | 100 | | | | 100 | | | 1 | | | 4 | |  |  |
| Multidisciplinary care assessment meetings | 100 | 100 | | | 0 | | | | 0 | | | 4 | | | 144 | |  |  |
| Safety monitoring | 0 | 0 | | | 100 | | | | 100 | | | 7 | | | 142 | |  |  |
| Exchange of information about health care and/or health services | 100 | 100 | | | 0 | | | | 0 | | | 399 | | | 1365 | |  |  |
| Health surveillance | 0 | 0 | | | 100 | | | | 100 | | | 7 | | | 35 | |  |  |
| Home visits | 100 | 100 | | | 0 | | | | 0 | | | 11 | | | 225 | |  |  |
| Associated activities | 0 | 0 | | | 100 | | | | 100 | | | 108 | | | 275 | |  |  |
| Support with exams/procedures | 0 | 0 | | | 100 | | | | 100 | | | 5 | | | 60 | |  |  |
| Dental consutation | 60,81 | 68,07 | | | 39,18 | | | | 31,92 | | | 444 | | | 10831 | |  |  |
| Health campaign | 0 | 0 | | | 100 | | | | 100 | | | 56 | | | 166 | |  |  |
| Scientific research | 0 | 0 | | | 100 | | | | 100 | | | 13 | | | 500 | |  |  |
| Care to spontaneous demand | 0 | 0 | | | 100 | | | | 100 | | | 2 | | | 20 | |  |  |
| **Rate for all activities with both modalities** | **39,26** | **49,73** | | | **60,73** | | | | **50,26** | | | **1808** | | | **21335** | |  |  |
| **Rate of the main activity** | **60,81** | **68,07** | | |  | | | |  | | |  | | |  | |  |  |
| **Professional category** | **Nurse** | | | | | | | | | | | | | | | |  |  |
|  | **Activities** | | | **Individual practice** | | | | | | | |  | | | | |  |  |
| **Activities** | **TR*** | **TR**** | | | **TR*** | | | | **TR**** | | | **Total*** | | | **Total**** | |  |  |
| Educational actions for health care professional (permanent training) | 0 | 0 | | | 100 | | | | 100 | | | 24 | | | 620 | |  |  |
| Infection control | 0 | 0 | | | 100 | | | | 100 | | | 89 | | | 155 | |  |  |
| Supply control | 3,57 | 1,73 | | | 96,42 | | | | 98,26 | | | 56 | | | 519 | |  |  |
| Urgent/emergency care | 0 | 0 | | | 100 | | | | 100 | | | 1 | | | 9 | |  |  |
| Community health development | 0 | 0 | | | 100 | | | | 100 | | | 3 | | | 98 | |  |  |
| Development of care processes and | 12,50 | 19,32 | | | 87,5 | | | | 80,67 | | | 8 | | | 326 | |  |  |
| Dispensation of medicines | 0 | 0 | | | 100 | | | | 100 | | | 2 | | | 21 | |  |  |
| Documentation | 0 | 0 | | | 100 | | | | 100 | | | 687 | | | 6135 | |  |  |
| Risk identification | 0 | 0 | | | 100 | | | | 100 | | | 2 | | | 45 | |  |  |
| Interpretation of laboratory data | 29,41 | 26,75 | | | 70,58 | | | | 73,24 | | | 17 | | | 157 | |  |  |
| Medicine administration | 0 | 0 | | | 100 | | | | 100 | | | 4 | | | 26 | |  |  |
| Vital Signs Measurement | 0 | 0 | | | 100 | | | | 100 | | | 6 | | | 39 | |  |  |
| Anthropometric Measurements | 0 | 0 | | | 100 | | | | 100 | | | 1 | | | 2 | |  |  |
| Organization of the work process | 0,68 | 0,45 | | | 99,31 | | | | 99,54 | | | 584 | | | 3290 | |  |  |
| Advice on the Health Care Sytem | 0 | 0 | | | 100 | | | | 100 | | | 222 | | | 504 | |  |  |
| Outpatient Procedures | 0 | 0 | | | 100 | | | | 100 | | | 100 | | | 2020 | |  |  |
| Promotion of education activities | 16,66 | 2,8 | | | 83,33 | | | | 97,2 | | | 6 | | | 250 | |  |  |
| Peripheral venipuncture | 0 | 0 | | | 100 | | | | 100 | | | 67 | | | 424 | |  |  |
| Administrative meetings | 100 | 100 | | | 0 | | | | 0 | | | 20 | | | 1124 | |  |  |
| Student support | 0 | 0 | | | 100 | | | | 100 | | | 80 | | | 659 | |  |  |
| Multidisciplinary care assessment meetings | 100 | 100 | | | 0 | | | | 0 | | | 22 | | | 967 | |  |  |
| Supervision of unit workers | 16,66 | 22,09 | | | 83,33 | | | | 77,90 | | | 12 | | | 86 | |  |  |
| Safety monitoring | 0 | 0 | | | 100 | | | | 100 | | | 10 | | | 160 | |  |  |
| Interagency transportation | 0 | 0 | | | 100 | | | | 100 | | | 6 | | | 81 | |  |  |
| Exchange of information about health care and/or health services | 100 | 100 | | | 0 | | | | 0 | | | 1950 | | | 5376 | |  |  |
| Health surveillance | 0 | 0 | | | 100 | | | | 100 | | | 51 | | | 859 | |  |  |
| Home visits | 93,54 | 94,88 | | | 6,45 | | | | 5,11 | | | 62 | | | 782 | |  |  |
| Associated activities | 1,65 | 1,37 | | | 98,34 | | | | 98,62 | | | 723 | | | 1596 | |  |  |
| Support with exams/procedures | 0 | 0 | | | 100 | | | | 100 | | | 59 | | | 357 | |  |  |
| Consutation | 9,13 | 15,16 | | | 90,86 | | | | 84,83 | | | 569 | | | 9137 | |  |  |
| Care to spontaneous demand | 0 | 0 | | | 100 | | | | 100 | | | 356 | | | 2360 | |  |  |
| Immunization control/Vaccionation | 0 | 0 | | | 100 | | | | 100 | | | 634 | | | 2011 | |  |  |
| **Rate for all activities with both modalities** | **33,09** | **24,31** | | | **66,90** | | | | **75,68** | | | **6433** | | | **40195** | |  |  |
| **Rate of the main activity** | **9,13** | **15,16** | | |  | | | |  | | |  | | |  | |  |  |
| **Professional Activities** | **Pharmacist** | | | | | | | | | | | | | | | |  |  |
|  | **Shared practice** | | | | | | **Individual practice** | | | |  | | | | | |  |  |
| **Activities** | **TR*** | **TR**** | | | **TR*** | | | | **TR**** | | | **Total*** | | | **Total**** | |  |  |
| Educational actions for health care professional (permanent training) | 0 | 0 | | | 100 | | | | 100 | | | 1 | | | 12 | |  |  |
| Infection control | 0 | 0 | | | 100 | | | | 100 | | | 1 | | | 1 | |  |  |
| Supply control | 0 | 0 | | | 100 | | | | 100 | | | 6 | | | 180 | |  |  |
| Development of care processes and | 0 | 0 | | | 100 | | | | 100 | | | 1 | | | 28 | |  |  |
| Dispensation of medicines | 0 | 0 | | | 100 | | | | 100 | | | 6 | | | 26 | |  |  |
| Documentation | 0 | 0 | | | 100 | | | | 100 | | | 6 | | | 132 | |  |  |
| Organization of the work process | 0 | 0 | | | 100 | | | | 100 | | | 11 | | | 120 | |  |  |
| Administrative meetings | 100 | 100 | | | 0 | | | | 0 | | | 1 | | | 118 | |  |  |
| Safety monitoring | 0 | 0 | | | 100 | | | | 100 | | | 3 | | | 43 | |  |  |
| Exchange of information about health care and/or health services | 100 | 100 | | | 0 | | | | 0 | | | 26 | | | 66 | |  |  |
| Associated activities | 0 | 0 | | | 100 | | | | 100 | | | 11 | | | 29 | |  |  |
| Consutation | 0 | 0 | | | 100 | | | | 100 | | | 2 | | | 29 | |  |  |
| **Rate for all activities with both modalities** | **36** | **23,47** | | | **64** | | | | **76,53** | | | **75** | | | **784** | |  |  |
| **Rate of the main activity** | **0** | **0** | | | **0** | | | |  | | |  | | |  | |  |  |
| **Profissional Activities** | **Physiotherapist** | | | | | | | | | | | | | | | |  |  |
|  | **Shared practice** | | | **Individual practice** | | | | | | |  | | | | | |  |  |
| **Activities** | **TR*** | **TR**** | | | **TR*** | | | | **TR**** | | | **Total*** | | | **Total**** | |  |  |
| Educational actions for health care professional (permanent training) | 0 | 0 | | |  | | | |  | | | 1 | | | 1 | |  |  |
| Infection control | 0 | 0 | | |  | | | |  | | | 8 | | | 8 | |  |  |
| Documentation | 0 | 0 | | |  | | | |  | | | 5 | | | 19 | |  |  |
| Organization of the work process | 0 | 0 | | |  | | | |  | | | 26 | | | 57 | |  |  |
| Advice on the Health Care Sytem | 0 | 0 | | |  | | | |  | | | 5 | | | 6 | |  |  |
| Exchange of information about health care and/or health services | 100 | 100 | | |  | | | |  | | | 35 | | | 121 | |  |  |
| Associated activities | 0 | 0 | | |  | | | |  | | | 31 | | | 40 | |  |  |
| Consutation | 0 | 0 | | |  | | | |  | | | 17 | | | 475 | |  |  |
| **Rate for all activities with both modalities** | **27,34** | **16,64** | | |  | | | |  | | | **128** | | | **727** | |  |  |
| **Rate of the main activity** | **0** | **0** | | |  | | | |  | | |  | | |  | |  |  |
| **Profissional Activities** | **Speech therapist** | | | | | | | | | | | | | | | |  |  |
|  | **Shared practice** | | | **Individual practice** | | | | | | |  | | | | | |  |  |
| **Activities** | **TR*** | **TR**** | | | **TR*** | | | | **TR**** | | | **Total*** | | | **Total**** | |  |  |
| Educational actions for health care professional (permanent training) | 0 | 0 | | | 100 | | | | 100 | | | 2 | | | 440 | |  |  |
| Infection control | 0 | 0 | | | 100 | | | | 100 | | | 1 | | | 1 | |  |  |
| Community health development | 0 | 0 | | | 100 | | | | 100 | | | 1 | | | 83 | |  |  |
| Documentation | 0 | 0 | | | 100 | | | | 100 | | | 25 | | | 372 | |  |  |
| Organization of the work process | 0 | 0 | | | 100 | | | | 100 | | | 19 | | | 141 | |  |  |
| Advice on the Health Care Sytem | 0 | 0 | | | 100 | | | | 100 | | | 5 | | | 12 | |  |  |
| Promotion of education activities | 0 | 0 | | | 100 | | | | 100 | | | 1 | | | 57 | |  |  |
| Administrative meetings | 100 | 100 | | | 0 | | | | 0 | | | 2 | | | 207 | |  |  |
| Multidisciplinary care assessment meetings | 100 | 100 | | | 0 | | | | 0 | | | 3 | | | 137 | |  |  |
| Exchange of information about health care and/or health services | 100 | 100 | | | 0 | | | | 0 | | | 70 | | | 423 | |  |  |
| Home visits | 28,57 | 42,72 | | | 71,42 | | | | 57,27 | | | 7 | | | 110 | |  |  |
| Associated activities | 0 | 0 | | | 100 | | | | 100 | | | 32 | | | 48 | |  |  |
| Consutation | 0 | 0 | | | 100 | | | | 100 | | | 9 | | | 289 | |  |  |
| **Rate for all activities with both modalities** | **43,50** | **35,08** | | | **56,49** | | | | **64,91** | | | **177** | | | **2320** | |  |  |
| **Rate of the main activity** | **0** | **0** | | |  | | | |  | | |  | | |  | |  |  |
| **Profissional Activities** | **Doctor** | | | | | | | | | | | | | | | |  |  |
|  | **Shared practice** | | | | | | | **Individual practice** | | |  | | | | | |  |  |
| **Activities** | **TR*** | **TR**** | | | **TR*** | | | | **TR**** | | | **Total*** | | | **Total**** | |  |  |
| Educational actions for health care professional (permanent training) | 0 | 0 | | | 100 | | | | 100 | | | 2 | | | 121 | |  |  |
| Infection control | 0 | 0 | | | 100 | | | | 100 | | | 80 | | | 114 | |  |  |
| Urgent/emergency care | 0 | 0 | | | 100 | | | | 100 | | | 9 | | | 77 | |  |  |
| Development of care processes and | 0 | 0 | | | 100 | | | | 100 | | | 3 | | | 162 | |  |  |
| Documentation | 0 | 0 | | | 100 | | | | 100 | | | 446 | | | 1835 | |  |  |
| Risk identification | 0 | 0 | | | 100 | | | | 100 | | | 3 | | | 49 | |  |  |
| Interpretation of laboratory data | 27,27 | 4 | | | 72,72 | | | | 96 | | | 11 | | | 150 | |  |  |
| Organization of the work process | 0,32 | 0,44 | | | 99,67 | | | | 99,55 | | | 308 | | | 896 | |  |  |
| Advice on the Health Care Sytem | 0 | 0 | | | 100 | | | | 100 | | | 45 | | | 100 | |  |  |
| Outpatient Procedures | 0 | 0 | | | 100 | | | | 100 | | | 8 | | | 99 | |  |  |
| Promotion of education activities | 0 | 0 | | | 100 | | | | 100 | | | 1 | | | 49 | |  |  |
| Reference and cross-reference | 0 | 0 | | | 100 | | | | 100 | | | 5 | | | 36 | |  |  |
| Administrative meetings | 100 | 100 | | | 0 | | | | 0 | | | 15 | | | 1127 | |  |  |
| Student support | 0 | 0 | | | 100 | | | | 100 | | | 160 | | | 1234 | |  |  |
| Multidisciplinary care assessment meetings | 100 | 100 | | | 0 | | | | 0 | | | 7 | | | 378 | |  |  |
| Safety monitoring | 0 | 0 | | | 100 | | | | 100 | | | 1 | | | 47 | |  |  |
| Interagency transportation | 0 | 0 | | | 100 | | | | 100 | | | 1 | | | 3 | |  |  |
| Exchange of information about health care and/or health services | 100 | 100 | | | 0 | | | | 0 | | | 661 | | | 1623 | |  |  |
| Health surveillance | 0 | 0 | | | 100 | | | | 100 | | | 2 | | | 17 | |  |  |
| Home visits | 100 | 100 | | | 0 | | | | 0 | | | 41 | | | 537 | |  |  |
| Associated activities | 1,76 | 1,93 | | | 98,23 | | | | 98,06 | | | 284 | | | 518 | |  |  |
| Support with exams/procedures | 0 | 0 | | | 100 | | | | 100 | | | 4 | | | 17 | |  |  |
| Consutation | 5,14 | 6,38 | | | 94,85 | | | | 93,61 | | | 700 | | | 9571 | |  |  |
| Care to spontaneous demand | 0 | 0 | | | 100 | | | | 100 | | | 44 | | | 451 | |  |  |
| **Rate for all activities with both modalities** | **27,06** | **22,36** | | | **72,93** | | | | **77,63** | | | **2841** | | | **19211** | |  |  |
| **Rate of the main activity** | **5,14** | **6,38** | | |  | | | |  | | |  | | |  | |  |  |
| **Profissional Activities** | **Nutricionist** | | | | | | | | | | | | | | | |  |  |
|  | **Shared practice** | | | **Individual practice** | | | | | | |  | | | | | |  |  |
| **Activities** | **TR*** | **TR**** | | | **TR*** | | | | **TR**** | | | **Total*** | | | **Total**** | |  |  |
| Educational actions for health care professional (permanent training) | 0 | 0 | | | 100 | | | | 100 | | | 1 | | | 600 | |  |  |
| Infection control | 0 | 0 | | | 100 | | | | 100 | | | 1 | | | 1 | |  |  |
| Community health development | 0 | 0 | | | 100 | | | | 100 | | | 2 | | | 173 | |  |  |
| Documentation | 0 | 0 | | | 100 | | | | 100 | | | 34 | | | 403 | |  |  |
| Risk identification | 0 | 0 | | | 100 | | | | 100 | | | 1 | | | 4 | |  |  |
| Organization of the work process | 0 | 0 | | | 100 | | | | 100 | | | 34 | | | 257 | |  |  |
| Advice on the Health Care Sytem | 0 | 0 | | | 100 | | | | 100 | | | 5 | | | 22 | |  |  |
| Reference and cross-reference | 0 | 0 | | | 100 | | | | 100 | | | 1 | | | 11 | |  |  |
| Administrative meetings | 100 | 100 | | | 0 | | | | 0 | | | 4 | | | 365 | |  |  |
| Multidisciplinary care assessment meetings | 100 | 100 | | | 0 | | | | 0 | | | 4 | | | 191 | |  |  |
| Supervision of unit workers | 0 | 0 | | | 100 | | | | 100 | | | 1 | | | 17 | |  |  |
| Exchange of information about health care and/or health services | 100 | 100 | | | 0 | | | | 0 | | | 47 | | | 251 | |  |  |
| Home visits | 100 | 100 | | | 0 | | | | 0 | | | 6 | | | 96 | |  |  |
| Associated activities | 0 | 0 | | | 100 | | | | 100 | | | 28 | | | 83 | |  |  |
| Performance evolution | 0 | 0 | | | 100 | | | | 100 | | | 1 | | | 29 | |  |  |
| Scientific research | 0 | 0 | | | 100 | | | | 100 | | | 1 | | | 45 | |  |  |
| Consutation | 0 | 0 | | | 100 | | | | 100 | | | 11 | | | 451 | |  |  |
| **Rate for all activities with both modalities** | **33,51** | **30,11** | | | **66,48** | | | | **69,88** | | | **182** | | | **2999** | |  |  |
| **Rate of the main activity** | **0** | **0** | | |  | | | |  | | |  | | |  | |  |  |
| **Profissional Activities** | **Psychologist** | | | | | | | | | | | | | | | |  |  |
|  | **Shared practice** | | | **Individual practice** | | | | | | |  | | | | | | |  |
| **Activities** | **TR*** | **TR**** | | | **TR*** | | | | **TR**** | | | **Total*** | | | **Total**** | |  |  |
| Infection control | 0 | 0 | | | 100 | | | | 100 | | | 3 | | | 3 | |  |  |
| Community health development | 50 | 87,20 | | | 50 | | | | 12,79 | | | 2 | | | 86 | |  |  |
| Development of care processes and | 0 | 0 | | | 100 | | | | 100 | | | 1 | | | 48 | |  |  |
| Documentation | 0 | 0 | | | 100 | | | | 100 | | | 28 | | | 333 | |  |  |
| Organization of the work process | 5,88 | 8,06 | | | 94,11 | | | | 91,93 | | | 51 | | | 186 | |  |  |
| Advice on the Health Care Sytem | 0 | 0 | | | 100 | | | | 100 | | | 3 | | | 8 | |  |  |
| Administrative meetings | 100 | 100 | | | 0 | | | | 0 | | | 3 | | | 415 | |  |  |
| Multidisciplinary care assessment meetings | 100 | 100 | | | 0 | | | | 0 | | | 10 | | | 525 | |  |  |
| Supervision of unit workers | 0 | 0 | | | 100 | | | | 100 | | | 1 | | | 44 | |  |  |
| Interagency transportation | 0 | 0 | | | 100 | | | | 100 | | | 1 | | | 33 | |  |  |
| Exchange of information about health care and/or health services | 100 | 100 | | | 0 | | | | 0 | | | 106 | | | 396 | |  |  |
| Health surveillance | 16,66 | 16,36 | | | 83,33 | | | | 83,63 | | | 6 | | | 55 | |  |  |
| Home visits | 66,66 | 95,91 | | | 33,33 | | | | 4,08 | | | 3 | | | 49 | |  |  |
| Associated activities | 2,63 | 1,33 | | | 97,36 | | | | 98,66 | | | 38 | | | 75 | |  |  |
| Scientific research | 0 | 0 | | | 100 | | | | 100 | | | 1 | | | 20 | |  |  |
| Consutation | 10,52 | 10,78 | | | 89,47 | | | | 89,21 | | | 19 | | | 547 | |  |  |
| Care to spontaneous demand | 0 | 0 | | | 100 | | | | 100 | | | 5 | | | 17 | |  |  |
| **Rate for all activities with both modalities** | **45,90** | **54,29** | | | **54,09** | | | | **45,70** | | | **281** | | | **2840** | |  |  |
| **Rate of the main activity** | **10,52** | **10,78** | | |  | | | |  | | |  | | |  | |  |  |
| **Profissional Activities** | **Psychiatrist** | | | | | | | | | | | | | | | |  |  |
|  | **Shared practice** | | | **Individual practice** | | | | | | |  | | | | | |  |  |
| **Activities** | **TR*** | **TR**** | | | **TR*** | | | | **TR**** | | | **Total*** | | | **Total**** | |  |  |
| Documentation | 0 | 0 | | | 100 | | | | 100 | | | 11 | | | 111 | |  |  |
| Organization of the work process | 0 | 0 | | | 100 | | | | 100 | | | 1 | | | 9 | |  |  |
| Advice on the Health Care Sytem | 0 | 0 | | | 100 | | | | 100 | | | 1 | | | 2 | |  |  |
| Exchange of information about health care and/or health services | 100 | 100 | | | 0 | | | | 0 | | | 7 | | | 41 | |  |  |
| Consutation | 0 | 0 | | | 100 | | | | 100 | | | 15 | | | 360 | |  |  |
| **Rate for all activities with both modalities** | **20** | **7,83** | | | **80** | | | | **92,16** | | | **35** | | | **523** | |  |  |
| **Rate of the main activity** | **0** | **0** | | | **0** | | | | **0** | | |  | | |  | |  |  |
| **Profissional Activities** | **Occupational Therapist** | | | | | | | | | | | | | | | |  |  |
|  | **Shared practice** | | | **Individual practice** | | | | | | |  | | | | | |  |  |
| **Activities** | **TR*** | **TR**** | | | **TR*** | | | | **TR**** | | | **Total*** | | | **Total**** | |  |  |
| Documentation | 0 | 0 | | | 100 | | | | 100 | | | 5 | | | 93 | |  |  |
| Administrative meetings | 100 | 100 | | | 0 | | | | 0 | | | 2 | | | 213 | |  |  |
| Exchange of information about health care and/or health services | 100 | 100 | | | 0 | | | | 0 | | | 2 | | | 23 | |  |  |
| Home visits | 66,66 | 80,59 | | | 33,33 | | | | 19,40 | | | 3 | | | 67 | |  |  |
| **Rate for all activities with both modalities** | **50** | **73,23** | | | **50** | | | | **26,76** | | | **12** | | | **396** | |  |  |
| **Rate of the main activity** | **0** | **0** | | | **0** | | | |  | | |  | | |  | |  |  |

**Table Legend:**

**Subtitle:** TR* Total rate based on the frequency of the activity;
 TR** Total rate based on the time of the activity;
